# Triethyl­ammonium 1,3-dimethyl-5-(2,4,6-trinitro­phenyl)barbiturate

**DOI:** 10.1107/S1600536812030450

**Published:** 2012-07-10

**Authors:** Kulandaiya Rajamani, Doraisamyraja Kalaivani

**Affiliations:** a PG and Research Department of Chemistry, Seethalakshmi Ramaswami College, Tiruchirappalli 620 002, Tamil Nadu, India

## Abstract

In the title mol­ecular salt [systematic name: triethyl­ammonium 1,3-dimethyl-2,6-dioxo-5-(2,4,6-trinitro­phen­yl)-1,2,3,6-tetra­hydro­pyrimidin-4-olate], C_6_H_16_N^+^·C_12_H_8_N_5_O_9_^−^, the dihedral angle between the aromatic rings in the anion is 46.88 (8)°. The nitro group *para* to the ring junction is almost coplanar with its attached ring [dihedral angle = 0.76 (3)°], but the two *ortho*-nitro groups are substanti­ally twisted from the ring plane, by 47.91 (2) and 42.90 (1)°. In the crystal, the cation and anion are linked by an N—H⋯O=C hydrogen bond; these dimeric associations are further connected by weak C—H⋯O bonds to form linear supra­molecular chains extending in the [001] direction.

## Related literature

For background to barbiturates, see: Tripathi (2009 ▶). For our recent work in this area, see: Kalaivani & Buvaneswari, 2010 ▶); Kalaivani & Malarvizhi (2009 ▶); Buvaneswari & Kalaivani (2011 ▶); Babykala & Kalaivani (2012 ▶).
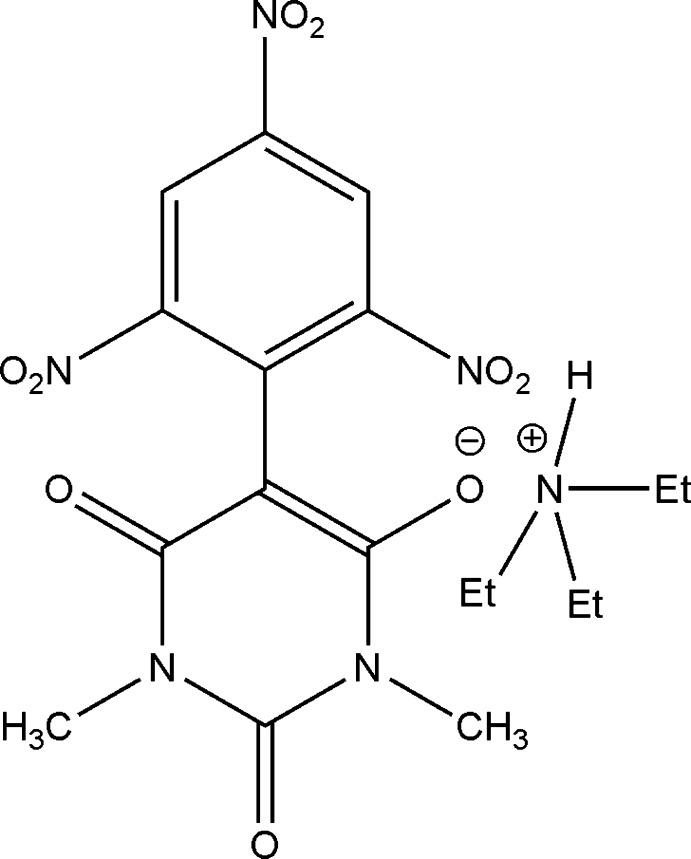


## Experimental

### 

#### Crystal data

C_6_H_16_N^+^·C_12_H_8_N_5_O_9_^−^
*M* *_r_* = 468.43
Monoclinic, 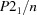

*a* = 10.967 (5) Å
*b* = 20.301 (5) Å
*c* = 11.072 (5) Å
β = 119.268 (5)°
*V* = 2150.4 (15) Å^3^
*Z* = 4
Mo *K*α radiation
μ = 0.12 mm^−1^
*T* = 293 K
0.30 × 0.20 × 0.20 mm

#### Data collection

Bruker Kappa APEXII CCD diffractometer
Absorption correction: multi-scan (*SADABS*; Bruker, 1999 ▶) *T*_min_ = 0.941, *T*_max_ = 0.988
18535 measured reflections
3785 independent reflections
2955 reflections with *I* > 2σ(*I*)
*R*_int_ = 0.029

#### Refinement

*R*[*F* ^2^ > 2σ(*F* ^2^)] = 0.036
*wR*(*F* ^2^) = 0.108
*S* = 1.01
3785 reflections
304 parameters
H-atom parameters constrained
Δρ_max_ = 0.28 e Å^−3^
Δρ_min_ = −0.17 e Å^−3^

### 

Data collection: *APEX2* (Bruker, 2004 ▶); cell refinement: *SAINT* (Bruker, 2004 ▶); data reduction: *SAINT*; program(s) used to solve structure: *SIR92* (Altomare *et al.*, 1993 ▶); program(s) used to refine structure: *SHELXL97* (Sheldrick, 2008 ▶); molecular graphics: *ORTEP-3* (Farrugia, 1997 ▶) and *Mercury* (Macrae *et al.*, 2008 ▶); software used to prepare material for publication: *PLATON* (Spek, 2009 ▶).

## Supplementary Material

Crystal structure: contains datablock(s) global, I. DOI: 10.1107/S1600536812030450/hb6883sup1.cif

Structure factors: contains datablock(s) I. DOI: 10.1107/S1600536812030450/hb6883Isup2.hkl

Supplementary material file. DOI: 10.1107/S1600536812030450/hb6883Isup3.cml

Additional supplementary materials:  crystallographic information; 3D view; checkCIF report

## Figures and Tables

**Table 1:** Hydrogen-bond geometry (Å, °)

*D*—H⋯*A*	*D*—H	H⋯*A*	*D*⋯*A*	*D*—H⋯*A*
N6—H6⋯O9	0.91	2.09	2.903 (2)	148
C13—H13*B*⋯O7^i^	0.97	2.37	3.195 (3)	143
C14—H14*B*⋯O3^ii^	0.96	2.55	3.301 (3)	136
C15—H15*A*⋯O8^iii^	0.97	2.52	3.275 (3)	134
C15—H15*B*⋯O8^iv^	0.97	2.49	3.437 (3)	165

Symmetry codes: (i) 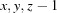; (ii) 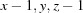; (iii) 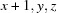; (iv) 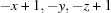.

**Fig. 1.**
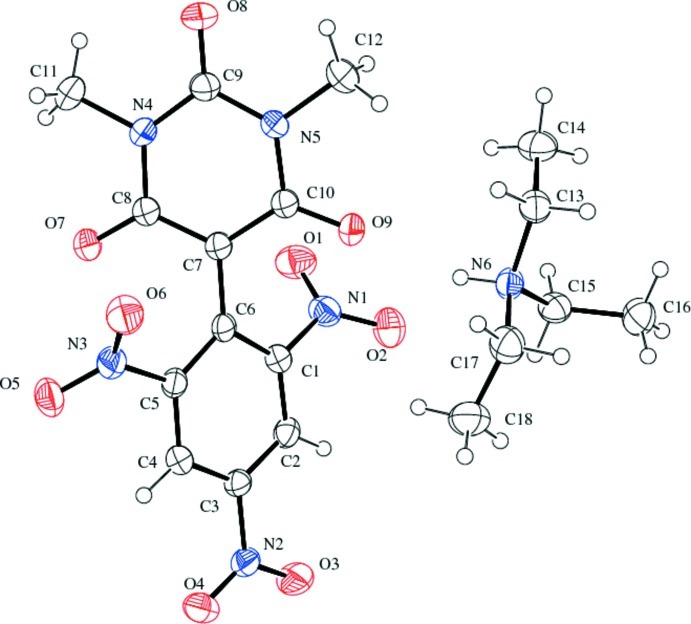
The asymmetric unit of the title compound showing 40% probability displacement ellipsoids.

**Fig. 2.**
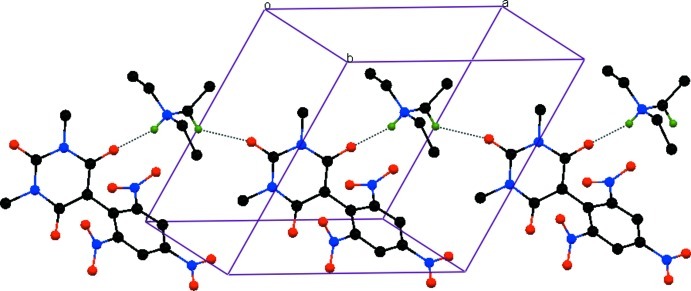
Packing view of title compound.

## supplementary crystallographic information

### Comment

Barbiturates are pyrimidine derivatives and many of them are anticonvulsant agents (Tripathi, 2009). The crystalline barbiturate (Triethylammonium 5-(2,4,6-trinitrophenyl)barbiturate) reported by us (Kalaivani & Buvaneswari, 2010) also exhibits anticovulsant activity and is soluble in water.An attempt has now been made to increase the lipophilicity of the above mentioned barbiturate by introducing methyl groups to the nitrogen atoms of barbiturate anion (Scheme). Since the N—H protons of barbiturate residue are replaced by methyl groups, *R*^2^_2_(8) motifs which have been normally observed in reported barbiturates (Kalaivani & Malarvizhi, 2009; Buvaneswari & Kalaivani, 2011; Babykala & Kalaivani, 2012), are absent in the crystal structure of the title barbiturate. The *ORTEP* view of the asymmetric unit of title molecule showing 40% probability displacement ellipsoids is shown in Fig.1. Weak N—H···O and C—H···O hydrogen bonds are noticed in the crystal structure which lead to supramolecular chains and these chains run along [001]direction [Fig.2]. In the title molecule, the two rings present in the anion moiety are not planar and the dihedral angle between them is 46.88 (8)°. The phenyl ring constituted by C1, C2, C3, C4, C5 and C6 carbon atoms and the nitro group with N2, O3 and O4 atoms are almost planar [dihedral angle 0.76 (3)°] which facilitates the delocalization of negative charge and thus the title molecular salt appears red in colour. The other two nitro groups (O1—N1—O2 and O5—N3—O6) make the dihedral angle 47.91 (2)° and 42.90 (1)° respectively with the aromatic ring.

### Experimental

Picrylchloride (1.3 g, 0.005 mol) was dissolved in 15 ml of absolute alcohol. 1,3-Dimethylbarbituric acid (0.8 g, 0.005 mol) was also dissolved in 15 ml of absolute alcohol.These two solution were mixed and to this 3 ml(0.03 mol) of analar grade triethylamine was added and shaken well for 6hrs. On standing maroon red crystals come out from this solution after 15 days. The crystals were filterded,powder well and washed with 30 ml of dry ether and recrystallized from absolute alcohol (yield of pure crystals 75%; m.pt; 458 K). Red block like single crystals suitable for X-ray studies were obtained by slow evapouration of ethanol at room temperature. The crystals obtained were non-hygroscopic and extraordinarly stable at room temperature. Solubility at 298 K: 19 g / dm^3^ (H_2_O); 46 g / dm^3^ (EtOH); 131 g / dm^3^ (DMSO);6 g /dm^3^ (n-Octanol).

### Refinement

Refinement of F^2^ against ALL reflections. The weighted *R*-factor *wR* and goodness of fit S are based on F^2^, conventional *R*-factors *R* are based on F, with F set to zero for negative F^2^. The threshold expression of F^2^ > 2sigma(F^2^) is used only for calculating *R*-factors(gt) *etc*. and is not relevant to the choice of reflections for refinement.

### Crystal data

**Table:**

C_6_H_16_N^+^·C_12_H_8_N_5_O_9_^−^	*F*(000) = 984
*M**_r_* = 468.43	*D*_x_ = 1.447 Mg m^−^^3^
Monoclinic, *P*2_1_/*n*	Mo *K*α radiation, λ = 0.71073 Å
*a* = 10.967 (5) Å	Cell parameters from 6627 reflections
*b* = 20.301 (5) Å	θ = 2.9–26.1°
*c* = 11.072 (5) Å	µ = 0.12 mm^−^^1^
β = 119.268 (5)°	*T* = 293 K
*V* = 2150.4 (15) Å^3^	Block, red
*Z* = 4	0.30 × 0.20 × 0.20 mm

### Data collection

**Table:**

Bruker Kappa APEXII CCD diffractometer	3785 independent reflections
Radiation source: fine-focus sealed tube	2955 reflections with *I* > 2σ(*I*)
Graphite monochromator	*R*_int_ = 0.029
ω and φ scan	θ_max_ = 25.0°, θ_min_ = 2.1°
Absorption correction: multi-scan (*SADABS*; Bruker, 1999)	*h* = −13→12
*T*_min_ = 0.941, *T*_max_ = 0.988	*k* = −24→24
18535 measured reflections	*l* = −13→13

### Refinement

**Table:**

Refinement on *F*^2^	Secondary atom site location: difference Fourier map
Least-squares matrix: full	Hydrogen site location: inferred from neighbouring sites
*R*[*F*^2^ > 2σ(*F*^2^)] = 0.036	H-atom parameters constrained
*wR*(*F*^2^) = 0.108	*w* = 1/[σ^2^(*F*_o_^2^) + (0.0548*P*)^2^ + 0.6716*P*] where *P* = (*F*_o_^2^ + 2*F*_c_^2^)/3
*S* = 1.01	(Δ/σ)_max_ = 0.001
3785 reflections	Δρ_max_ = 0.28 e Å^−^^3^
304 parameters	Δρ_min_ = −0.17 e Å^−^^3^
0 restraints	Extinction correction: *SHELXL97* (Sheldrick, 2008), Fc^*^=kFc[1+0.001xFc^2^λ^3^/sin(2θ)]^-1/4^
Primary atom site location: structure-invariant direct methods	Extinction coefficient: 0.0019 (6)

### Special details

**Table:**

Geometry. All e.s.d.'s (except the e.s.d. in the dihedral angle between two l.s. planes) are estimated using the full covariance matrix. The cell e.s.d.'s are taken into account individually in the estimation of e.s.d.'s in distances, angles and torsion angles; correlations between e.s.d.'s in cell parameters are only used when they are defined by crystal symmetry. An approximate (isotropic) treatment of cell e.s.d.'s is used for estimating e.s.d.'s involving l.s. planes.
Refinement. Refinement of *F*^2^ against ALL reflections. The weighted *R*-factor *wR* and goodness of fit *S* are based on *F*^2^, conventional *R*-factors *R* are based on *F*, with *F* set to zero for negative *F*^2^. The threshold expression of *F*^2^ > σ(*F*^2^) is used only for calculating *R*-factors(gt) *etc*. and is not relevant to the choice of reflections for refinement. *R*-factors based on *F*^2^ are statistically about twice as large as those based on *F*, and *R*- factors based on ALL data will be even larger.

### Fractional atomic coordinates and isotropic or equivalent isotropic displacement parameters (Å^2^)

**Table:**

	*x*	*y*	*z*	*U*_iso_*/*U*_eq_	
C1	0.88311 (17)	0.10654 (8)	0.91121 (17)	0.0341 (4)	
C2	1.02255 (18)	0.12113 (9)	0.98506 (17)	0.0362 (4)	
H2	1.0876	0.0978	0.9710	0.043*	
C3	1.06380 (16)	0.17129 (9)	1.08071 (16)	0.0335 (4)	
C4	0.96883 (17)	0.20600 (9)	1.10282 (17)	0.0354 (4)	
H4	0.9979	0.2388	1.1700	0.042*	
C5	0.82951 (17)	0.19082 (8)	1.02269 (16)	0.0330 (4)	
C6	0.77688 (17)	0.14087 (8)	0.92164 (16)	0.0318 (4)	
C7	0.62923 (17)	0.12556 (9)	0.83656 (16)	0.0345 (4)	
C8	0.54703 (18)	0.11973 (9)	0.90224 (17)	0.0378 (4)	
C9	0.34306 (18)	0.09753 (9)	0.67628 (17)	0.0369 (4)	
C10	0.57272 (17)	0.11594 (8)	0.69361 (17)	0.0344 (4)	
C11	0.3184 (2)	0.09483 (14)	0.8813 (2)	0.0681 (7)	
H11A	0.2885	0.1368	0.8971	0.102*	
H11B	0.3712	0.0723	0.9679	0.102*	
H11C	0.2381	0.0689	0.8211	0.102*	
C12	0.3617 (2)	0.09733 (12)	0.46746 (19)	0.0578 (6)	
H12A	0.3738	0.0528	0.4458	0.087*	
H12B	0.4041	0.1271	0.4313	0.087*	
H12C	0.2638	0.1070	0.4266	0.087*	
C13	0.63642 (18)	0.12726 (9)	0.33536 (18)	0.0409 (4)	
H13A	0.5743	0.1558	0.3506	0.049*	
H13B	0.6456	0.1453	0.2591	0.049*	
C14	0.5725 (2)	0.06017 (10)	0.2963 (2)	0.0527 (5)	
H14A	0.6219	0.0345	0.2612	0.079*	
H14B	0.4762	0.0641	0.2263	0.079*	
H14C	0.5783	0.0388	0.3763	0.079*	
C15	0.87815 (18)	0.07822 (9)	0.45773 (18)	0.0420 (4)	
H15A	0.9655	0.0800	0.5444	0.050*	
H15B	0.8402	0.0342	0.4487	0.050*	
C16	0.9074 (2)	0.09033 (12)	0.3404 (2)	0.0554 (5)	
H16A	0.8216	0.0880	0.2541	0.083*	
H16B	0.9710	0.0575	0.3418	0.083*	
H16C	0.9481	0.1332	0.3504	0.083*	
C17	0.8343 (2)	0.19556 (10)	0.4964 (2)	0.0514 (5)	
H17A	0.8565	0.2100	0.4259	0.062*	
H17B	0.7625	0.2246	0.4930	0.062*	
C18	0.9621 (2)	0.20199 (12)	0.6351 (2)	0.0648 (6)	
H18A	0.9427	0.1854	0.7052	0.097*	
H18B	0.9885	0.2475	0.6530	0.097*	
H18C	1.0371	0.1772	0.6360	0.097*	
N1	0.85117 (16)	0.04632 (8)	0.82617 (16)	0.0453 (4)	
N2	1.21193 (15)	0.18730 (8)	1.16185 (15)	0.0395 (4)	
N3	0.73551 (15)	0.23498 (7)	1.04510 (15)	0.0397 (4)	
N4	0.40581 (14)	0.10460 (8)	0.81685 (14)	0.0403 (4)	
N5	0.42801 (14)	0.10512 (7)	0.61771 (14)	0.0373 (4)	
N6	0.77754 (14)	0.12699 (7)	0.46335 (14)	0.0370 (3)	
H6	0.7634	0.1141	0.5343	0.044*	
O1	0.77641 (16)	0.00505 (7)	0.83646 (16)	0.0609 (4)	
O2	0.90781 (17)	0.03998 (8)	0.75520 (16)	0.0690 (5)	
O3	1.29408 (13)	0.15560 (8)	1.14104 (15)	0.0588 (4)	
O4	1.24725 (14)	0.23153 (8)	1.24621 (15)	0.0605 (4)	
O5	0.77284 (14)	0.25027 (7)	1.16504 (13)	0.0534 (4)	
O6	0.63133 (14)	0.25617 (7)	0.94531 (14)	0.0532 (4)	
O7	0.59132 (14)	0.12529 (8)	1.02758 (13)	0.0545 (4)	
O8	0.21962 (12)	0.08442 (7)	0.60642 (13)	0.0507 (4)	
O9	0.63886 (13)	0.11793 (7)	0.62877 (12)	0.0470 (3)	

### Atomic displacement parameters (Å^2^)

**Table:**

	*U*^11^	*U*^22^	*U*^33^	*U*^12^	*U*^13^	*U*^23^
C1	0.0351 (9)	0.0377 (9)	0.0296 (9)	−0.0007 (7)	0.0159 (7)	−0.0015 (7)
C2	0.0339 (9)	0.0426 (10)	0.0369 (9)	0.0050 (7)	0.0209 (8)	0.0019 (8)
C3	0.0277 (8)	0.0425 (10)	0.0299 (8)	0.0000 (7)	0.0137 (7)	0.0021 (7)
C4	0.0359 (9)	0.0407 (10)	0.0294 (9)	−0.0018 (7)	0.0160 (7)	−0.0026 (7)
C5	0.0326 (9)	0.0397 (10)	0.0303 (9)	0.0024 (7)	0.0183 (7)	0.0007 (7)
C6	0.0331 (9)	0.0404 (9)	0.0245 (8)	−0.0003 (7)	0.0160 (7)	0.0039 (7)
C7	0.0305 (9)	0.0446 (10)	0.0288 (9)	−0.0012 (7)	0.0147 (7)	0.0001 (7)
C8	0.0336 (9)	0.0498 (11)	0.0305 (9)	−0.0024 (8)	0.0161 (8)	−0.0021 (7)
C9	0.0310 (10)	0.0413 (10)	0.0356 (9)	0.0009 (7)	0.0143 (8)	−0.0021 (7)
C10	0.0328 (9)	0.0406 (10)	0.0301 (9)	−0.0002 (7)	0.0157 (8)	0.0008 (7)
C11	0.0449 (12)	0.119 (2)	0.0525 (13)	−0.0154 (12)	0.0337 (11)	−0.0137 (12)
C12	0.0460 (12)	0.0905 (17)	0.0299 (10)	−0.0027 (11)	0.0132 (9)	−0.0005 (10)
C13	0.0357 (10)	0.0494 (11)	0.0360 (10)	0.0086 (8)	0.0162 (8)	0.0042 (8)
C14	0.0371 (10)	0.0581 (13)	0.0529 (12)	0.0011 (9)	0.0142 (9)	−0.0018 (10)
C15	0.0344 (10)	0.0454 (11)	0.0410 (10)	0.0055 (8)	0.0145 (8)	0.0017 (8)
C16	0.0458 (12)	0.0736 (15)	0.0521 (12)	0.0104 (10)	0.0281 (10)	−0.0039 (10)
C17	0.0601 (13)	0.0454 (11)	0.0546 (12)	−0.0036 (9)	0.0327 (11)	−0.0063 (9)
C18	0.0546 (13)	0.0687 (15)	0.0713 (15)	−0.0167 (11)	0.0310 (12)	−0.0205 (12)
N1	0.0412 (9)	0.0464 (10)	0.0439 (9)	0.0012 (7)	0.0173 (8)	−0.0074 (7)
N2	0.0323 (8)	0.0496 (9)	0.0372 (8)	−0.0013 (7)	0.0176 (7)	0.0010 (7)
N3	0.0360 (8)	0.0470 (9)	0.0385 (9)	0.0034 (7)	0.0202 (7)	−0.0026 (7)
N4	0.0307 (8)	0.0596 (10)	0.0345 (8)	−0.0039 (7)	0.0190 (7)	−0.0033 (7)
N5	0.0305 (8)	0.0527 (9)	0.0261 (7)	−0.0019 (6)	0.0118 (6)	−0.0007 (6)
N6	0.0377 (8)	0.0433 (8)	0.0336 (8)	0.0024 (6)	0.0203 (7)	0.0031 (6)
O1	0.0591 (9)	0.0453 (8)	0.0744 (10)	−0.0100 (7)	0.0295 (8)	−0.0083 (7)
O2	0.0717 (10)	0.0792 (11)	0.0714 (10)	−0.0022 (8)	0.0470 (9)	−0.0295 (8)
O3	0.0326 (7)	0.0740 (10)	0.0692 (10)	0.0027 (7)	0.0244 (7)	−0.0134 (8)
O4	0.0431 (8)	0.0759 (10)	0.0599 (9)	−0.0148 (7)	0.0232 (7)	−0.0280 (8)
O5	0.0512 (8)	0.0702 (10)	0.0411 (8)	0.0074 (7)	0.0244 (6)	−0.0139 (7)
O6	0.0412 (8)	0.0656 (9)	0.0472 (8)	0.0187 (6)	0.0172 (7)	0.0046 (7)
O7	0.0452 (8)	0.0924 (11)	0.0289 (7)	−0.0134 (7)	0.0205 (6)	−0.0051 (6)
O8	0.0282 (7)	0.0692 (9)	0.0474 (8)	−0.0049 (6)	0.0127 (6)	−0.0074 (6)
O9	0.0398 (7)	0.0745 (9)	0.0324 (7)	−0.0049 (6)	0.0220 (6)	−0.0027 (6)

### Geometric parameters (Å, º)

**Table:**

C1—C2	1.368 (2)	C13—C14	1.496 (3)
C1—C6	1.410 (2)	C13—N6	1.503 (2)
C1—N1	1.477 (2)	C13—H13A	0.9700
C2—C3	1.376 (2)	C13—H13B	0.9700
C2—H2	0.9300	C14—H14A	0.9600
C3—C4	1.375 (2)	C14—H14B	0.9600
C3—N2	1.458 (2)	C14—H14C	0.9600
C4—C5	1.375 (2)	C15—C16	1.502 (3)
C4—H4	0.9300	C15—N6	1.506 (2)
C5—C6	1.408 (2)	C15—H15A	0.9700
C5—N3	1.476 (2)	C15—H15B	0.9700
C6—C7	1.454 (2)	C16—H16A	0.9600
C7—C10	1.403 (2)	C16—H16B	0.9600
C7—C8	1.413 (2)	C16—H16C	0.9600
C8—O7	1.231 (2)	C17—C18	1.494 (3)
C8—N4	1.398 (2)	C17—N6	1.496 (2)
C9—O8	1.216 (2)	C17—H17A	0.9700
C9—N4	1.367 (2)	C17—H17B	0.9700
C9—N5	1.379 (2)	C18—H18A	0.9600
C10—O9	1.246 (2)	C18—H18B	0.9600
C10—N5	1.403 (2)	C18—H18C	0.9600
C11—N4	1.461 (2)	N1—O1	1.216 (2)
C11—H11A	0.9600	N1—O2	1.224 (2)
C11—H11B	0.9600	N2—O4	1.214 (2)
C11—H11C	0.9600	N2—O3	1.2174 (19)
C12—N5	1.462 (2)	N3—O6	1.2153 (19)
C12—H12A	0.9600	N3—O5	1.2231 (19)
C12—H12B	0.9600	N6—H6	0.9100
C12—H12C	0.9600		
			
C2—C1—C6	124.81 (16)	H14A—C14—H14B	109.5
C2—C1—N1	114.05 (15)	C13—C14—H14C	109.5
C6—C1—N1	120.89 (15)	H14A—C14—H14C	109.5
C1—C2—C3	118.09 (15)	H14B—C14—H14C	109.5
C1—C2—H2	121.0	C16—C15—N6	113.47 (15)
C3—C2—H2	121.0	C16—C15—H15A	108.9
C4—C3—C2	121.56 (15)	N6—C15—H15A	108.9
C4—C3—N2	119.27 (15)	C16—C15—H15B	108.9
C2—C3—N2	119.16 (15)	N6—C15—H15B	108.9
C3—C4—C5	118.02 (16)	H15A—C15—H15B	107.7
C3—C4—H4	121.0	C15—C16—H16A	109.5
C5—C4—H4	121.0	C15—C16—H16B	109.5
C4—C5—C6	124.66 (15)	H16A—C16—H16B	109.5
C4—C5—N3	113.74 (15)	C15—C16—H16C	109.5
C6—C5—N3	121.49 (14)	H16A—C16—H16C	109.5
C5—C6—C1	112.77 (15)	H16B—C16—H16C	109.5
C5—C6—C7	124.26 (15)	C18—C17—N6	113.69 (18)
C1—C6—C7	122.98 (15)	C18—C17—H17A	108.8
C10—C7—C8	121.86 (15)	N6—C17—H17A	108.8
C10—C7—C6	119.90 (15)	C18—C17—H17B	108.8
C8—C7—C6	118.23 (15)	N6—C17—H17B	108.8
O7—C8—N4	118.59 (15)	H17A—C17—H17B	107.7
O7—C8—C7	124.97 (16)	C17—C18—H18A	109.5
N4—C8—C7	116.41 (15)	C17—C18—H18B	109.5
O8—C9—N4	122.11 (16)	H18A—C18—H18B	109.5
O8—C9—N5	121.55 (16)	C17—C18—H18C	109.5
N4—C9—N5	116.33 (15)	H18A—C18—H18C	109.5
O9—C10—C7	125.81 (16)	H18B—C18—H18C	109.5
O9—C10—N5	117.82 (15)	O1—N1—O2	124.41 (17)
C7—C10—N5	116.35 (15)	O1—N1—C1	118.11 (16)
N4—C11—H11A	109.5	O2—N1—C1	117.37 (16)
N4—C11—H11B	109.5	O4—N2—O3	123.31 (15)
H11A—C11—H11B	109.5	O4—N2—C3	118.63 (15)
N4—C11—H11C	109.5	O3—N2—C3	118.05 (15)
H11A—C11—H11C	109.5	O6—N3—O5	124.33 (15)
H11B—C11—H11C	109.5	O6—N3—C5	119.06 (14)
N5—C12—H12A	109.5	O5—N3—C5	116.51 (14)
N5—C12—H12B	109.5	C9—N4—C8	124.58 (14)
H12A—C12—H12B	109.5	C9—N4—C11	117.14 (15)
N5—C12—H12C	109.5	C8—N4—C11	118.28 (15)
H12A—C12—H12C	109.5	C9—N5—C10	124.20 (14)
H12B—C12—H12C	109.5	C9—N5—C12	116.65 (15)
C14—C13—N6	112.98 (15)	C10—N5—C12	119.06 (15)
C14—C13—H13A	109.0	C17—N6—C13	109.94 (14)
N6—C13—H13A	109.0	C17—N6—C15	113.33 (14)
C14—C13—H13B	109.0	C13—N6—C15	113.64 (14)
N6—C13—H13B	109.0	C17—N6—H6	106.5
H13A—C13—H13B	107.8	C13—N6—H6	106.5
C13—C14—H14A	109.5	C15—N6—H6	106.5
C13—C14—H14B	109.5		
			
C6—C1—C2—C3	2.7 (3)	C6—C1—N1—O2	138.16 (18)
N1—C1—C2—C3	−171.73 (15)	C4—C3—N2—O4	−0.7 (2)
C1—C2—C3—C4	0.2 (3)	C2—C3—N2—O4	−179.81 (16)
C1—C2—C3—N2	179.31 (15)	C4—C3—N2—O3	179.52 (16)
C2—C3—C4—C5	−2.3 (3)	C2—C3—N2—O3	0.4 (2)
N2—C3—C4—C5	178.59 (15)	C4—C5—N3—O6	135.01 (17)
C3—C4—C5—C6	1.8 (3)	C6—C5—N3—O6	−41.4 (2)
C3—C4—C5—N3	−174.45 (15)	C4—C5—N3—O5	−41.5 (2)
C4—C5—C6—C1	0.7 (2)	C6—C5—N3—O5	142.10 (16)
N3—C5—C6—C1	176.70 (14)	O8—C9—N4—C8	−179.90 (17)
C4—C5—C6—C7	−179.45 (16)	N5—C9—N4—C8	1.0 (3)
N3—C5—C6—C7	−3.4 (2)	O8—C9—N4—C11	0.8 (3)
C2—C1—C6—C5	−3.0 (2)	N5—C9—N4—C11	−178.25 (18)
N1—C1—C6—C5	171.01 (15)	O7—C8—N4—C9	179.50 (17)
C2—C1—C6—C7	177.13 (16)	C7—C8—N4—C9	−2.3 (3)
N1—C1—C6—C7	−8.8 (2)	O7—C8—N4—C11	−1.2 (3)
C5—C6—C7—C10	134.21 (17)	C7—C8—N4—C11	176.98 (18)
C1—C6—C7—C10	−46.0 (2)	O8—C9—N5—C10	−175.62 (17)
C5—C6—C7—C8	−46.9 (2)	N4—C9—N5—C10	3.4 (2)
C1—C6—C7—C8	132.98 (18)	O8—C9—N5—C12	0.7 (3)
C10—C7—C8—O7	177.32 (18)	N4—C9—N5—C12	179.73 (17)
C6—C7—C8—O7	−1.6 (3)	O9—C10—N5—C9	175.68 (16)
C10—C7—C8—N4	−0.7 (3)	C7—C10—N5—C9	−6.2 (2)
C6—C7—C8—N4	−179.65 (16)	O9—C10—N5—C12	−0.5 (2)
C8—C7—C10—O9	−177.38 (17)	C7—C10—N5—C12	177.61 (17)
C6—C7—C10—O9	1.5 (3)	C18—C17—N6—C13	169.79 (16)
C8—C7—C10—N5	4.7 (2)	C18—C17—N6—C15	−61.8 (2)
C6—C7—C10—N5	−176.45 (15)	C14—C13—N6—C17	−179.66 (16)
C2—C1—N1—O1	129.19 (17)	C14—C13—N6—C15	52.1 (2)
C6—C1—N1—O1	−45.4 (2)	C16—C15—N6—C17	−66.4 (2)
C2—C1—N1—O2	−47.2 (2)	C16—C15—N6—C13	60.1 (2)

### Hydrogen-bond geometry (Å, º)

**Table:**

*D*—H···*A*	*D*—H	H···*A*	*D*···*A*	*D*—H···*A*
N6—H6···O9	0.91	2.09	2.903 (2)	148
C13—H13*B*···O7^i^	0.97	2.37	3.195 (3)	143
C14—H14*B*···O3^ii^	0.96	2.55	3.301 (3)	136
C15—H15*A*···O8^iii^	0.97	2.52	3.275 (3)	134
C15—H15*B*···O8^iv^	0.97	2.49	3.437 (3)	165

Symmetry codes: (i) *x*, *y*, *z*−1; (ii) *x*−1, *y*, *z*−1; (iii) *x*+1, *y*, *z*; (iv) −*x*+1, −*y*, −*z*+1.
